# Exploring the gut microbiota: lifestyle choices, disease associations, and personal genomics

**DOI:** 10.3389/fnut.2023.1225120

**Published:** 2023-10-05

**Authors:** Sharlize Pedroza Matute, Sasitaran Iyavoo

**Affiliations:** ^1^Nkaarco Diagnostics Limited, Norwich, United Kingdom; ^2^School of Chemistry, College of Health and Science, University of Lincoln, Lincoln, United Kingdom

**Keywords:** gut, microbiota, microbiome, lifestyle, nutrition, exercise, genomics, dysbiosis

## Abstract

The gut microbiota is a rich and dynamic ecosystem that actively interacts with the human body, playing a significant role in the state of health and disease of the host. Diet, exercise, mental health, and other factors have exhibited the ability to influence the gut bacterial composition, leading to changes that can prevent and improve, or favor and worsen, both intestinal and extra-intestinal conditions. Altered gut microbial states, or ‘dysbiosis’, associated with conditions and diseases are often characterized by shifts in bacterial abundance and diversity, including an impaired *Firmicutes* to *Bacteroidetes* ratio. By understanding the effect of lifestyle on the gut microbiota, personalized advice can be generated to suit each individual profile and foster the adoption of lifestyle changes that can both prevent and ameliorate dysbiosis. The delivery of effective and reliable advice, however, depends not only on the available research and current understanding of the topic, but also on the methods used to assess individuals and to discover the associations, which can introduce bias at multiple stages. The aim of this review is to summarize how human gut microbial variability is defined and what lifestyle choices and diseases have shown association with gut bacterial composition. Furthermore, popular methods to investigate the human gut microbiota are outlined, with a focus on the possible bias caused by the lack of use of standardized methods. Finally, an overview of the current state of personalized advice based on gut microbiota testing is presented, underlining its power and limitations.

## 1. Introduction

The human gut is inhabited by an estimated 10^14^ microorganisms, represented by a mixture of bacteria, archaea, fungi, and protozoa that start colonizing the gut at birth and evolve with the hosts throughout their lifespan. This population of organisms is referred to as the ‘gut microbiota’ and it represents the result of thousands of years of coevolution and mutualistic relationship with the human body (1). The genomic content of the gut microbiota, termed ‘gut microbiome’, accommodates at least 100 times the genetic content of the human genome, and with the recent advances in technologies like DNA sequencing, it provides a window into the composition of the gut ecosystem (2).

By contributing to host nutrient metabolism, protection from pathogens, host immunity regulation, and maintenance of a healthy intestinal epithelium, the gut microbiota has emerged as a key-player in multiple aspects of host health (3–5). The gut microbiota synthesizes vitamins such as vitamin K and most water-soluble vitamins B, including biotin, cobalamin, folates, nicotinic acid, pantothenic acid, pyridoxine, riboflavin, and thiamine (6, 7). It also produces neurotransmitters and hormones that function as signals to the central nervous system and can affect mood and cognition (8, 9). The human gut microbiota is also involved in the degradation and fermentation of fibers and the biotransformation of bile acids produced by the liver, promoting nutrients absorption and metabolism, and therefore influencing the overall state of health of the host (10–12). States of altered microbial composition called ‘dysbiosis’ have been associated with multiple disorders, including inflammatory bowel disease (IBD), inflammatory bowel syndrome (IBS), obesity, diabetes, asthma, psoriasis, cancer, and neurological conditions (13, 14).

Regardless of the level of conservation of the human gut microbiota composition, shaped by millennia of mutualistic coevolution and natural selection, daily lifestyle choices, including diet and exercise, have revealed to play a role in the relative abundance of gut bacteria. This, in turn, signifies that individuals have the power to adapt their lifestyle to prevent or improve states of dysbiosis (1, 15, 16).

Given the effect of dysbiosis in human health, improving knowledge around gut bacteria and their function opens the way to gut microbial testing and personalized lifestyle advice aimed at preventing diseases and promoting health. As a result, multiple companies have started offering gut microbial tests to a wider population. However, to effectively deliver gut-microbiome-based advice and have control over interventions and their outcome, clarity is required on what can affect the gut microbiota and how its composition is defined, allowing accurate testing and formulation of personalized advice based on scientific evidence.

This review aims at summarizing the present knowledge around human gut microbial variability, its association with health and disease and the effect exerted by lifestyle factors, with a focus on diet, xenobiotics, exercise, and mental health. The most popular available methods to define gut microbiota composition are also highlighted, taking into consideration pros and cons and the current lack of standardization. Finally, this review offers an overview of the current status of personalized advice based on gut microbial testing, underlining its power and limitations, as well as ethical issues.

## 2. Gut microbial composition

The analysis of gut microbiome studies revealed that 90% of the phyla found in humans belong to *Bacteroidetes* and *Firmicutes*, with *Actinobacteria*, *Proteobacteria*, *Fusobacteria*, and *Verrucomicrobia* representing a large part of the remaining 10%. Most of the *Firmicutes* are gram-positive bacteria and are represented by *Clostridium* (95%)*, Lactobacillus, Bacillus, Enterococcus,* and *Ruminicoccus*, while *Bacteroidetes* are gram-negative bacteria mainly constituted by *Bacteroides* and *Prevotella*. On the other hand, the *Bifidobacterium* genus is the main representative of the *Actinobacteria* (5, 17).

Individual gut microbial composition can be influenced during infancy by factors such as being born preterm or full term, by vaginal delivery or caesarean, and being fed human milk (18–20). It also varies with the age, from childhood (up to 1 year old) to adulthood and over 70, and it can be greatly affected by antibiotic treatments, Body Mass Index (BMI), exercise, and diet (5, 18, 21, 22). Metagenomic data from healthy individuals with different ancestry also showed that the impact of diet and environment on the gut microbial composition and function is greater than the influence of inherited genes (23).

Wide variation in microbial composition between individuals has been highlighted by important metagenomic projects such as the Human Microbiome Project (HMP) and the European Metagenomics of the Human Intestinal Tract project (MetaHIT) (24, 25). Attempts were made to define a basic gut microbiome profile including a collection of microbes present in all individuals (common core), although more similarities were identified from a genetic and therefore functional point of view rather than taxonomic (26, 27). Data from a cohort of 124 European individuals (healthy, overweight, or with IBD) revealed 18 species present in all the individuals and 57 in more than 90% of the participants, with inter-individual variability from ~10 to ~2000-fold due to high variation in microbial abundance. The same study showed that, regardless of bacterial variability, common identified functions were related to genes involved in bacterial fitness in the gut and genes important for interaction with the host, including degradation of complex polysaccharides and synthesis of short chain fatty acids, indispensable amino acids, and vitamins (25).

Further studies allowed the identification of clusters of microbiome variation that indicate the presence of a limited number of balanced microbial states, termed ‘enterotypes’. The analysis of variability of the gut microbiota composition and function in 33 individuals identified three distinct clusters in which one of three genera has different abundance, with *Bacteroides* dominant in enterotype I, *Prevotella* in enterotype II and *Ruminococcus* in enterotype III. Comparison with other studies revealed similar clustering, especially for enterotypes I and II, with a third group driven by *Clostridiales*-related groups. Strong correlations between the distribution of the dominant genera and the other genera present in the same individual were also observed. In the same investigation, a deeper analysis of the enterotypes functional profiles highlighted that different enterotypes may rely on different routes to generate energy (fermentation from carbohydrates for enterotype I, from mucin glycoproteins for II and III) and show different level of vitamins production (17).

The existence of enterotypes has however been questioned as other studies found that most of the available data seem to support the presence of continuous gradients of variation rather than simplified discrete enterotypes (28, 29). Associating an enterotype cluster to a disease could lead to loss of perspective on intra-cluster variation compared to using predictive methods based on taxon-relative abundance. Machine learning-based classifiers performed better when classifying, for example, lean adults versus individuals with obesity and healthy people versus patients with Crohn’s disease (27, 29, 30). Another limitation of the concept of enterotypes is represented by the assumption that an individual gut microbiota composition is stable during adulthood, while data suggest that healthy subjects’ microbiomes can, over time, vary as much as the variability observed between different individuals (17, 29). This variation can be due to, among others, diseases, dietary changes, the use of medications, and other lifestyle changes that will be discussed in more details further ahead in this review (31). Recent attempts to settle the debate around the validity of enterotypes included a meta-analysis of gut microbial composition data obtained from three large datasets (HMP, MetaHIT, and a Chinese study). It was found that groups of samples tend to cluster around preferred genus level compositions, resulting in some more frequent relative abundance profiles. However, this should not limit the pursuit of more complex analyses (24, 32–34). The importance of enterotypes is therefore highlighted as a way of capturing similar microbial patterns that may be relevant for clinical approaches and dietary interventions. However, better definition, improved methods, and standardization may be required to identify samples that do not fit in the defined clusters. A classifier developed from the abovementioned meta-analysis, to be used with caution, is available at [http://enterotypes.org] (accessed on 06/02/23) (32).

## 3. Lifestyle

### 3.1. Diet and gut microbiota

Among the factors capable of influencing the gut microbiota composition, diet appears to have a major effect, both in the short and long term. The analysis of fecal samples and dietary habits from 98 individuals revealed a strong association between protein and animal fat-rich diets with *Bacteroides*-driven microbiome profiles (enterotype I), and carbohydrate-rich diets with *Prevotella*-abundant profiles (enterotype II). Specifically, *Bacteroidetes* and *Actinobacteria* positively correlated with fat but negatively with fiber, while *Firmicutes* and *Proteobacteria* behaved in the opposite way. In the same study, a 10-day high-fat/low-fiber or low-fat/high-fiber monitored diet revealed a shift in microbiome composition as soon as 24 h after the beginning of the diet, but not enough to change enterotype or to match the inter-individual differences (35). Another study showed that an exclusively plant-based diet can rapidly alter human gut microbial composition and gene expression, with significant changes detected 2 days after the adoption of the diet and return to baseline composition 2 days after the end of the 5-day diet (21). As previously observed, *Prevotella* showed positive correlation with fiber intake, while a high-fat animal-based diet led to an increase in bile-resistant microorganisms like *Bilophila* and *Bacteroides*, recreating patterns observed in herbivorous and carnivorous mammals’ gut profiles (21, 35, 36). Rapid and reversible changes in gut bacterial composition were also observed in relation to diets high in resistant starch or non-starch polysaccharides and a reduced carbohydrate diet provided to overweight individuals, causing detectable changes as soon as 3–4 days after the adoption of the diet. In the same study, individuals on a diet high in resistant starch exhibited an increase in the percentage of *Ruminococcus bromii* and *Eubacterium rectale-*related bacteria, even if inter-individual differences were predominant (37).

The comparison of children from rural Africa and Europe showed a higher amount of the *Bacteroidetes* genera *Prevotella* and *Xylanibacter* in the African fiber-rich diet, compared to a higher amount of *Firmicutes* and *Enterobacteriaceae* bacteria found in European children, whose diet is more animal-based and richer in sugar (38). Carbohydrates that can be used as source of energy for gut microbes, known as microbiota-accessible carbohydrates (MACs), are mainly represented by dietary fibers/plant polysaccharides and they are abundant in the African diet. On the contrary, the Western diet, which is typically low in these carbohydrates (low-MACs diet), has been associated with lower microbial gene richness and decreased diversity (26, 39–42). Furthermore, African children’s fecal samples showed higher abundance of short chain fatty acids (SCFAs), which are the product of the fermentation of complex carbohydrate such as resistant starch and inulin (38, 43).

SCFAs, which include acetate, propionate, and butyrate, represent a source of energy for colonocytes, promoting intestinal motility and a healthy epithelial barrier. These fatty acids have also been associated with anti-inflammatory properties and inhibition of cancer cells growth (38, 43, 44). In addition, they have been shown to stimulate hormonal and neuronal signals involved with the suppression of appetite (45). The most investigated SCFA is butyrate, which can be obtained directly through consumption of food like butter and cheese or indirectly through bacterial fermentation of dietary fiber, fermentation of acetate and lactate produced by certain microbial species, or degradation of mucin carried out by some *Clostridia* species (46). Butyrate represents over 70% of the colonocytes energy source and impaired butyrate metabolism has been observed in pathologies such as colitis and colon cancer (44, 47, 48).

The consumption of single dietary components has also been associated with specific changes in gut microbial composition. A recent metagenomic sequencing of fecal samples obtained from more than 1,100 United Kingdom and United States of America (USA) individuals showed, among others, the following associations: full fat yoghurt consumption and increased *Bifidobacterium animalis* and *Streptococcus thermophilus*; coffee consumption and butyrate-producing *Lawsonibacter asaccharolyticus*; healthy plant-based food and butyrate-producing bacteria such as *Roseburia hominis, Agathobaculum butyriciproducens, Faecalibacterium prausnitzii,* and *Anaerostipes hadrus*; ‘less-healthy’ plant-based and animal-based foods and *Clostridium* species. The same study also showed that gut microbiome profiles of healthy animal-based diets cluster separately from diets involving less-healthy animal-based food (49).

An overview of specific nutrients and diets and their relationship with health and disease states is discussed in Section 5.

### 3.2. Environment and gut microbiota

#### 3.2.1. Geography and ethnicity

As emerged from the comparison between African and European children’s samples, the effect of diet on the gut microbiota is apparent when comparing gut signature profiles of people living in different countries. To provide another example, the comparison of the fecal microbiota composition of Korean and USA adult twin pairs revealed some signature biogeography, likely influenced by diet and other environmental factors, with significantly different bacterial compositions between the two countries. The same study showed greater differences, within each country, between the microbiota of individuals from different families rather than those from the same familial group, which could be due to similar habits, environment, and/or similar genetics. Moreover, lean American and Korean individuals showed greater microbial differences compared to obese individuals, highlighting weight and lifestyle choices as a confounding factor (50).

The analysis of fecal microbiota of 2,084 individuals with different ethnicities but living in the same cities highlighted ethnicity as an important factor influencing interindividual differences. In this context, even though the genetic component could determine the initial establishment of gut profiles, environmental factors, diet in particular, are expected to contribute to differences in a higher degree than genetics (51). In fact, a survey comparing the gut microbiome of 1,046 healthy individuals with different genetic ancestry but sharing a common environment showed a limited role of host genetics in determining the gut microbial composition (23).

Further evidence of the influence of the country of residence on gut microbiome diversity and function has been reported after observation of the effect of immigration on Thai individuals relocating to the USA. Migrating to the USA has been associated not only with an increase in metabolic disease and obesity rates, but also with rapid changes in the gut microbiota composition. These changes include loss of native gut microbial species, loss of diversity, and increase in *Bacteroides* at the expense of *Prevotella* strains, with progression following the time spent in the USA (52). The reasons behind this could be partially explained by cultural adaptation to a Western diet and increased sedentary lifestyle, and as a result of a history of food insecurity (53, 54).

#### 3.2.2. Living with pets

Among the environmental factors that might affect the gut microbiota, living with pets appears to play a role. The analysis of stool samples from 332 adult participants from households with or without pets revealed no differences in bacterial diversity but significant differences in the abundance of certain phyla, predominantly *Firmicutes* (55). Exposure to furry pets has also been linked to changes in gut microbiota profiles of infants, with an increase in *Ruminococcus* and *Oscillospira*, which have been associated with obesity and predisposition to allergies (56). However, it has also been hypothesized that exposure to pets in infants might be linked to allergy predisposition through modulation of gut microbiota, with preliminary data indicating a protective effect (57). Further research is therefore necessary to clarify the relationship between pets and gut bacteria.

### 3.3. Xenobiotics, alcohol, and gut microbiota

Among the substances that individuals are exposed to daily, drugs, environmental pollutants, and food additives should also be taken into consideration as potential modifiers of the gut microbiota composition. Overall, these foreign compounds are referred to as ‘xenobiotics’, which are voluntarily or involuntarily ingested by humans. Once in the intestine, the xenobiotics can interact with gut bacteria by affecting their growth and functions. Furthermore, they can be metabolized by the bacteria resulting in molecules with a different role, toxicity, and persistence than the initial compound (58–60). However, this interaction is quite complex and not yet fully understood (61).

#### 3.3.1. Prescription and recreational drugs

A plethora of host-targeted drugs are modified by gut bacteria, even if the implications and mechanisms are still not fully comprehended (62, 63). In this regard, a study comparing the effect of 6 host-targeted drugs and 8 antibiotics showed little impact of the host-targeted drugs on the microbial physiology. Antibiotics, however, caused significant differences in the structure of the microbial community, especially in the *Firmicutes* taxonomic group. Besides, the bacterial gene expression for drug resistance and metabolism changed significantly with both types of treatment (62). By either killing or inhibiting bacterial growth or reproduction, antibiotics can have a profound effect on the gut microbiota, causing perturbations that can have serious impact on the state of health of an individual. Antibiotics such as vancomycin, ampicillin, streptomycin, and metronidazole have all been associated with changes in the gut microbial composition, causing, for example, reduced microbial diversity and altered gram-positive over gram-negative ratio, with an increase in gram-negative species (64). The use of antibiotics can also lead to increased susceptibility to *Clostridium difficile* infection, which represents a major issue for hospitalized elderly patients. *C. difficile*, which forms spores that can persist in the environment, can thrive in the gut of patients with antibiotic-related dysbiosis and often causes reinfection, with mortality rates higher than 10% in patients over 80 years of age (65–67). On the other hand, treatments with the antibiotic rifaximin have been found to increase the levels of the beneficial bacteria *Bifidobacterium, Faecalibacterium prausnitzii,* and *Lactobacillus* (68). On a side note, it is relevant to mention that antibiotics may not always be ingested consciously. In fact, veterinary antibiotics administered to animals ultimately accumulate in both aquatic and terrestrial ecosystems. This occurs through various pathways, including, for example, soil fertilization, eventually leading to the inclusion of antibiotics in the food chain (69–72).

The use of recreational drugs, including marijuana, has also been linked to gut microbial changes, including dysbiosis (73). The *Prevotella*:*Bacteroides* ratio was found to be 13 times lower in chronic marijuana users than non-users, and this change appeared to be correlated with reduced cognitive functions. However, the possible impact of a different diet, low in fresh produce, on marijuana users, could have contributed to the differences reported in the study. Therefore, this association should be further investigated (74). On the other hand, preliminary studies have shown a potential anti-inflammatory effect of cannabidiol (CBD), a major cannabinoid found in cannabis, in the human gut (75). Studies in mice using CBD and/or tetrahydrocannabinol (THC), another primary cannabinoid, also showed reduced inflammation, amelioration of dysbiosis, increased in beneficial gut bacteria, and potential protection from, for example, diabetes, obesity, and cardiovascular conditions (76–79). The mechanism of action of cannabinoids is related to the activation of cannabinoid receptors expressed in the nervous system, including the enteric nervous system, in some immune cells, and in epithelial cells. In the gut, the activation of the endocannabinoid system has been linked to intestinal motility modulation, promotion of endothelial membrane integrity, and prevention of inflammation. The psychoactive effect of THC, and not of CBD, should, however, be kept in mind when evaluating the use of cannabis or cannabinoids for therapeutic purposes (80). Regardless of the expected positive impact, studies on the potential beneficial effects of CBD and THC on non-alcoholic fatty liver disease showed contrasting results, which highlight the need for better standardization. In fact, different cannabis variety may contain different numbers and amounts of bioactive compounds, potentially leading to different effects (81, 82). A systematic review and meta-analysis has also found inconsistent results regarding cannabis’ impact on the gut microbiome across studies, highlighting the need for more comprehensive research to decipher the precise mechanisms and consequences of cannabis on gut microbiota (83). Furthermore, cannabis consumption has demonstrated potential benefits in ulcerative colitis patients, such as altering endocannabinoid tone and inducing mucosal healing, but the involvement of gut microbiota in these effects remains unclear (84). Other recreational drugs, such as cocaine and methamphetamine, have been linked to negative changes in gut microbial composition (85, 86). However, more research is necessary to evaluate the full range of consequences of recreational drugs on gut health.

#### 3.3.2. Environmental pollutants

Environmental pollutants such as heavy metals and pesticides have also been shown to alter the gut microbiota composition, leading to dysbiosis and negative health effects (64, 87, 88). For example, lead, cadmium, and arsenic exposure in mice has been reported to cause a change in bacterial community structure and relative abundances, causing shifts in the *Firmicutes* to *Bacteroidetes* ratio (64, 89–91). Besides, gut bacteria offer not only a physical barrier to the absorption of heavy metals, but also provide enzymes that can contribute to the detoxification of these compounds. In particular, probiotics, live lactic acid bacteria such as *Lactobacillus* and *Bifidobacterium* contained in fermented food, have shown the ability to significantly reduce the toxic effect of heavy metals by limiting their absorption, providing detoxification, maintaining gut barrier integrity, and reducing the expression of metal transporters (92–94). Pesticides such as herbicides, insecticides, and fungicides are all being explored for their impact on the gut microbiota, generally revealing shifts in relative abundances in the model organisms used, but sometimes yielding contrasting results (95). More research is therefore needed in this field to clearly outline the functionality and impact of these compounds on intestinal bacteria.

#### 3.3.3. Food additives

Food additives, such as artificial sweeteners, emulsifiers, preservatives, and colorants are also being investigated to shed light on the possible impact on the gut microbial community structure. Artificial sweeteners such as aspartame, acesulfame, saccharine, sucralose, and cyclamate showed the capability to induce changes in the gut microbiota, reducing the bacterial diversity and impairing the glucose metabolism (96, 97). However, the dosage of sweeteners used in these studies does not always seem to represent the average daily intake in the normal population. Surveys on the Belgian population above 15 years of age and Irish pre-school children both showed an average daily intake of artificial sweeteners well below the acceptable daily intake (ADI), defined as the threshold below which no adverse effect on human health would be expected over a lifetime (98–100). Regardless, some studies may use a sweetener dosage that matches or surpasses the ADI. For example, a 2017 study on the effect of acesulfame potassium on mice showed shifts in gut bacterial composition after administration of 37.5 mg/kg body weight/day of the sweetener, which has an ADI of 15 mg/kg body weight/day (96, 101). Because of the variable dosage used in different studies, as well as the characteristic effect showed by each sweetener and the scarcity of investigations on humans, it is important to be cautious in the evaluation of the significance of these studies for human health. Emulsifiers have also appeared to influence the gut microbiota and have been linked to increased inflammation (15, 102). Little research has focused, to date, on the effect of preservatives and colorants on the gut microbiota, with initial data showing a certain degree of impact on the gut microbial community structure (96). However, further investigations are required to deepen our understanding of these compounds and their impact on gut microbiota and overall health.

#### 3.3.4. Cigarettes

Cigarettes and e-cigarettes smoking studies consistently revealed low gut bacterial diversity, while conflicting results were obtained in terms of what bacterial phyla or genus was more or less represented in the group of smokers compared to non-smokers, even if significant differences were often present (103). The mechanism of action is still unclear, but the multiple toxic substances contained in cigarette smoking are likely to play a role. Polycyclic aromatic hydrocarbons, aldehydes, nitrosamines, benzene, and heavy metals, among others, may reach the gut and act as antimicrobial, as well as change the pH, affect organic acids production, and be metabolized by gut bacteria creating further toxic compounds. These molecules may favor some bacterial species while negatively affecting others, leading to dysbiosis (103, 104). Nicotine itself has been linked to changes in gut microbial population structure, leading to an increase in *Proteobacteria* and *Bacteroidetes* phyla and a reduction in *Actinobacteria* and *Firmicutes* (105).

#### 3.3.5. Alcohol

Alcohol has also been identified as a cause of microbiota variation. Dysbiosis has been observed in individuals suffering from alcohol dependance, and the gut microbiota is also believed to play a role in the pathogenesis of alcoholic liver disease. Specifically, altered abundance of *Bacteroidetes* and *Enterococcaceae* has been observed in patients affected by alcoholic liver disease (106, 107). A study in mice revealed bacterial overgrowth, *Enterobacteriaceae* in particular, and intestinal inflammation after alcohol administration for 7 days, indicating an effect of alcohol even with acute rather than prolonged exposure (107). The mechanism causing alterations in the gut microbiota appears linked to the production of acetate: ethanol does not seem to be metabolized by gut bacteria, but instead an increase in acetate levels generated by host enzymes is likely to play a role (108). Given the link found between alcohol and gut bacteria, multiple studies have suggested that probiotics may be employed to improve the levels of liver-associated enzymes (109).

### 3.4. Physical exercise and gut microbiota

Physical exercise has shown potential to independently alter the composition and function of the gut microbiota. Exercise has been associated with increased butyrate production in animals and transient and reversible changes in the gut microbiome in humans (110–112). For instance, when compared to sedentary controls, athletes exhibited different gut microbiome profiles with increased diversity and different relative abundance, with elevated proportions of *Firmicutes* and lower amount of *Bacteroidetes*. However, the difference in dietary habits of athletic individuals compared to sedentary people, e.g., increased protein consumption, is likely to have played a role in the observed differences (113). In another study, pre-menopausal women performing at least 3 h of exercise a week showed higher levels of butyrate producers compared to women not reaching the same threshold of exercise, even though different diet regimens (e.g., significantly more fibers in the active group) might have had an impact on the results (114).

In the first controlled longitudinal study aimed at evaluating the independent effect of exercise on the gut microbiota, sedentary individuals, lean or with obesity, adopting consistent dietary patterns during a 6-week endurance training, faced differing transient gut microbiome and metabolic alterations: lean individuals experienced an increase in SCFAs production, increase in *Faecalibacterium*, and decrease in *Bacteroides*, while the microbiome of individuals with obesity shifted in the opposite direction (110). In another attempt to distinguish between the effect of diet and the effect of exercise, 24 previously sedentary men with the same nutritional profile were divided into exercise and control groups, with the exercise group following a 10-week moderate aerobic training. Results showed that the exercise-induced increase of oxygen uptake correlated with higher gut microbial diversity and higher relative abundance of *Roseburia*, *Sutterella*, and *Odoribacter* genera (115).

The intensity of exercise has also been linked to the gut microbiota composition: aerobic exercise on amateurs has been associated with improved gut microbial diversity and positive influence on gut health, while endurance, exhaustive exercise on athletes has been correlated with an increase in adverse effects, including reduced microbial diversity and elevated intestinal distress and inflammation (16). On another note, young people exercising daily exhibited increased gut microbial diversity, with higher levels of *Firmicutes*, suggesting that the frequency of exercise itself could also influence the gut microbiota composition (5, 116).

Among the mechanisms of interaction between exercise and gut microbiota, it has been proposed that communication mediated by the autonomic nervous system signals through the vagus nerve, neuroendocrine signaling through the hypothalamic–pituitary–adrenal axis, and serotonin regulation could play a role (117, 118). However, several other mechanisms have been suggested, including the effect of exercise on the intestinal integrity, permeability, and motility, and the effect of raised body temperature and reduced intestinal blood flow (111, 119–121). Intense exercise, in particular, can increase the permeability of the intestinal wall and reduce the thickness of the gut mucus, potentially allowing pathogens in the bloodstream and increasing systemic inflammation (122). This may also promote contact between gut bacteria and mucosal immune cells, affecting the microbial balance (111). Also, the altered gut motility resulting from exercise, including reduced transit time and improved gas movement, may induce physical changes in the gastrointestinal tract that affect pH, mucus secretion, biofilm formation, and nutrients availability, with a consequential effect on the bacterial composition (111, 123, 124).

### 3.5. Brain activity and gut microbiota

#### 3.5.1. Mental health

Psychological stressors, even with short exposure, displayed the ability to modify the gut microbiota composition in mice, causing a significant reduction in *Lactobacillus,* which presence has been associated with health benefits, including immunomodulation and reduction of inflammation (125–127). Besides, certain neuropeptides produced in the gut have displayed antimicrobial activity, while hormones such as adrenaline, noradrenaline, and cortisol produced through activation of the hypothalamic–pituitary–adrenal axis have been correlated with bacterial pathogen’s growth (128, 129). In addition, a study on mice revealed that the colonization of the gut with microbes can lead to an increased anxiety-like behavior compared to germ-free mice, indicating that the gut microbiota may contribute to regulate emotions (130). This is likely attributed to the bi-directional interaction between the central nervous system and the enteric nervous system via the vagal nerve, commonly referred to as the gut-brain axis. It could also be due to the neuropeptides generated by endocrine cells and the neurotransmitters potentially produced by gut bacteria (129, 131–133). Gut bacteria could also influence the central nervous system thorough SCFAs production. SCFAs have been shown to influence epigenetic regulation, which has been linked, in turn, to the development of brain and behavior (134). It is also believed that SCFAs may affect the permeability of the gut epithelium and reach the brain through the bloodstream, affecting it directly (135). Interestingly, another study on mice revealed that highly caloric diets rich in fat and sugar could be linked to changes in microbial composition that adversely affect mood (136).

The abundance of animal studies on the subject have helped identify the possible mechanisms involved in the correlation between mental health and gut microbiota in humans. Human studies have mainly focused on the administration of bacterial strains such as *Lactobacillus* and *Bifidobacterium*, showing improvement in depression, stress, and cognitive functions (137, 138). A study on students subjected to academic stress, in fact, revealed a reduction in fecal lactic acid bacteria during periods of high stress levels (139). Human studies also showed that anxiety and depression correlate with IBS symptoms severity, that depression caused by stressful events may be linked with increased abundance of *Enterobacteriaceae*, while psychological stress may be related to low levels of *Lactobacilli* spp. and higher levels of *Escherichia coli* and *Pseudomonas* spp. (140, 141). There is also evidence that stress and depression may increase the permeability of the gut barrier, leading to bacterial translocation into the blood stream and increased systemic inflammation (142, 143). The mechanism involved in stress-related increased permeability appears linked to the release of cortisol and the activation of mast cells via corticotropin-releasing hormone (144). Stress-induced preference for unhealthy food and metabolic changes triggered by stress and depression may also influence the gut microbiota through a change in diet and food digestion efficiency (142, 145, 146). Gut bacteria may, in turn, influence food choices through the production of molecules that resemble or interfere with peptides and hormones involved in appetite regulation, or by influencing mood and eating behavior via the gut-brain-axis (142, 147–149).

#### 3.5.2. Sleep and circadian rhythm

The gut-brain-axis has not only been associated with mental health disorders, but also with sleep regulation. Microbial metabolites, as well as the serotonergic system, the vagus nerve, and the immune system have all emerged as vehicles of communication between the gut and the brain involved in regulating sleep (150). Recent studies have revealed a daily rhythmicity in the composition of gut microbiota and relative metabolites, primarily influenced by feeding patterns, as well as other circadian cues, e.g., light/dark cycles (151, 152). While host circadian-rhythm parameters can influence the gut microbiota, gut bacteria may in turn modulate host rhythms through metabolites production (152). A disturbance of the equilibrium caused, for example, by traveling, has been linked to gut microbial changes likely caused by jet lag and sleep loss which impact the diurnal rhythms, and, consequently, the composition and function of gut microbes (153, 154). Dysbiotic gut microbial profiles have been observed in people affected by sleep disturbances, indicating the possible role of bacteria in the pathogenesis of such conditions, and opening the way to gut-microbiota-targeted solutions (155). Dietary supplementation including, for example, probiotics and vitamins, and increased attention to the time of feeding have the potential to improve maintaining the regularity of the circadian rhythm and consequently improve sleep (150–152).

## 4. Disease and gut microbiota

### 4.1. Intestinal disorders

The gut microbial composition and metabolic profiles have been extensively studied in relation to multiple conditions and diseases, from intestinal disorders to extra-intestinal illnesses (25, 156). It is generally accepted that the ratio between the two main phyla *Firmicutes* and *Bacteroidetes* represents an important indication of gut health, with a shift in this ratio being referred to as dysbiosis (157, 158). A decrease in obligate anaerobes and increase in facultative anaerobes including pathogens such as *E. coli, Salmonella, Proteus*, *Klebsiella*, and *Shigella* have also been highlighted as a common characteristic of dysbiosis in humans and animals (43).

Gut bacterial dysbiosis has been shown in both constipated and diarrheal patients (159, 160). Patients with IBS have shown significant microbiome shifts compared to healthy individuals, with lower diversity, increased *Firmicutes* to *Bacteroidetes* ratio, less *Lactobacilli*, *Faecalibacterium* and *Bifidobacteria* and more *Veillonella*, *Streptococci, Ruminococcus* spp. and *Enterobacteriaceae* (5, 140, 161). In IBD, including Crohn’s disease and ulcerative colitis, dysbiosis and reduced bacterial diversity have been reported, with lower numbers of *Firmicutes* and a relative increase of potentially pathogenic bacteria of the *Enterobacteriaceae* family, such as *Escherichia coli*, compared to commensals (25, 156, 162).

Celiac disease, an autoimmune intestinal condition triggered by dietary gluten, has also been linked to gut microbiota dysbiosis, with increased numbers of rod-shaped and gram-negative bacteria (163, 164). It is also hypothesized that the gut bacteria might contribute to the development of the disease by interacting with the same receptors responsible for the activation of innate immunity (165). The use of probiotics bears potential in reducing inflammation and improve symptoms in celiac disease, but further studies are required to better evaluate their therapeutic power (163).

Food allergies, including cow’s milk, peanuts, and eggs, have also been associated with dysbiosis, even if no specific bacterial taxa have so far been consistently identified in correlation to specific allergies. However, increasing evidence is supporting the role of dysbiosis in the pathogenetic process, especially during early-life gut colonization and immune development (166, 167). Use of antibiotics, for example, can affect gut microbial balance and increase the risk of allergies, as microbial molecules can affect oral tolerance by interacting with pattern recognition receptors on immune cells. On the other hand, SCFAs can protect from allergies by leading to an increase in T regulatory cells, by regulating the expression of enzymes involved in immune tolerance, and by reducing the production of pro-inflammatory cytokines (166, 168).

Lactose intolerance, which is thought to be caused by a combination of diet and the genetic profile of the individual, e.g., functional variation in the lactase gene, has also been linked to the gut levels of *Bifidobacterium* and other bacteria that perform lactose fermentation (169, 170). The ability of gut bacteria to ferment lactose influences the amount of lactose and its metabolites present in the intestine, contributing to the osmotic effects responsible for the symptoms (171). Abundance of *Bifidobacterium* has been observed in lactose intolerant individuals consuming dairy products and a positive correlation with symptoms has been established, suggesting a role of *Bifidobacterium* in mediating gastrointestinal effects of lactose intolerance (169). However, in another study, an increase in *Bifidobacterium* mediated by supplementation with purified short-chain galacto-oligosaccharides correlated with an improvement in lactose intolerance symptoms (172). These contrasting findings indicate that more investigations are required to evaluate the real impact of gut bacteria in mediating lactose intolerance symptoms.

Colorectal cancer has been associated with a reduction in butyrate-producing *Lachnospiraceae* bacteria and an enrichment in, for example, *Fusobacterium* spp., *Escherichia/Shigella*, *Klebsiella*, *Streptococcus*, *Peptostreptococcus*, and opportunistic pathogens (5, 173, 174). Gut dysbiosis has also been identified as potential trigger of colorectal cancer, contributing to its development and pathogenesis (175, 176). Other forms of gastrointestinal cancer, including gastric cancer, esophageal cancer, liver, and pancreatic cancer have been linked to the gut microbiota and their metabolites, which may contribute to carcinogenesis by triggering inflammation, altering the immune system regulation, and affecting pharmacodynamics (177, 178).

### 4.2. Extra-intestinal disorders

Intestinal disorders aside, multiple extra-intestinal disorders have also been associated with gut microbial dysbiosis, including metabolic disorders, autoimmune diseases, skin conditions, and central nervous system disorders (5, 179–181).

Obesity has been associated with altered *Bacteroidetes:Firmicutes* ratio, with greater relative abundance of *Firmicutes*, and with a higher proportion of genes related to SCFAs production, which underlines a higher capacity of obtaining energy from food (182, 183). However, there is also evidence of a shift in the *Bacteroidetes* to *Firmicutes* ratio in the opposite direction (184). Obesity has also been linked to the microbiota alterations caused by diet and antibiotics, to altered bile acid metabolism, to chronic inflammation and enrichment in specific bacterial groups such as *Prevotellaceae* and *Enterobacteriaceae* families, *Enterobacter* and *Roseburia* genera (185).

Type 2 diabetes has been typically linked to less *Firmicutes* and *Clostridia*, increased *Bacteroidetes* to *Firmicutes* ratio, less butyrate-producing bacteria and more sulfate-reducing bacteria as *Desulfovibrionaceae* spp. (5, 34, 186). Sulfate reducing bacteria, which levels are influenced by the availability of inorganic sulfates/sulfites and dietary amino acids and mucins, have also been associated with the onset of IBD, IBS, and colorectal cancer (187).

The gut microbiota composition and function have been identified as potential contributor to cardiovascular diseases, which include, among others, hypertension, coronary artery disease, and atherosclerosis. These are of prime relevance as they also represent the main cause of death in developed countries (188, 189). High blood pressure variability, a risk factor for cardiovascular incidents, has been linked to increased abundance of *Prevotella* spp. and *Clostridium* spp. and lower levels of *Alistipesfinegoldii* and *Lactobacillus* spp., indicating a possible involvement of the gut microbiota in the regulation of blood pressure (190). Positive correlation between high blood pressure and *Klebsiella* spp. and *Streptococcaceae* has also been shown, together with reduced microbial diversity (191, 192). These correlations may be explained by the anti-inflammatory effect linked to SCFAs production and the direct regulation of blood pressure by SCFAs through surface receptors. They could also be a result of the action of metabolites produced by the gut microbiota, which act as an endocrine organ both locally and on distant organs (188, 191, 193). Hyperlipidemia, characterized by elevated levels of total cholesterol, low-density lipoprotein cholesterol, and triglycerides, and a low level of high-density lipoprotein cholesterol, has also been associated with gut microbial composition. For example, individuals with hyperlipidemia have been observed having lower levels of SCFA-producing bacteria. This relationship could be explained by the gut microbiota ability to regulate the lipid metabolism in the host through production of metabolites including bile acids, SCFAs, and lipopolysaccharides (194). Atherosclerosis, a chronic inflammation of the arteries wall characterized by lipid accumulation, is linked to high levels of cholesterol in the blood and could therefore be linked to gut microbial composition, because of the above-mentioned capability of gut metabolites to influence blood lipid levels (195). Furthermore, certain bacteria that are predominant in the gut have been found in atherosclerotic plaques, indicating that these bacteria might be able to cross the intestinal barrier and reach the blood stream, where they can contribute to the development of atherosclerosis (196).

The metabolic syndrome is defined by a combination of physiological, biochemical, clinical, and metabolic factors linked to an increased risk of cardiovascular disease and type 2 diabetes. Among the factors that constitute the syndrome are visceral fat, hypertension, hyperlipidemia, and chronic inflammation often associated with obesity and diabetes (197). The metabolic syndrome has been linked to dysbiosis, characterized by low abundance of probiotics such as *Bifidobacterium*, *Lactobacillus*, and *Roseburia* and high abundance of lipopolysaccharides-producing bacteria (194, 198, 199). The production of a bacterial toxin such as lipopolysaccharide results in a local and systemic low-grade state of inflammation that in turn promotes obesity and insulin resistance (198, 200). As a result of this and the important role of the gut microbiota in, among others, host food digestion and energy harvest, epithelial homeostasis maintenance, and protection against pathogens, dysbiosis has been proposed as potential pathogenic contributor to the development of the disease (198). The chronic systemic inflammation that characterizes the metabolic syndrome has also been proposed as a possible promoter of osteoarthritis, indicating a potential correlation between gut microbial dysbiosis and this degenerative cartilage deterioration condition (201).

Gout, a condition characterized by elevated levels of uric acid, arthritis, and inflammation in the joints has been associated with high levels of *Prevotella*, *Fusobacterium*, and *Bacteroides* and low levels of *Enterobacteriaceae* and butyrate-producing bacteria (202, 203). These changes lead to a lower degradation of uric acid, increased uric acid production, and increased inflammation (202, 204). Gut microbial dysbiosis, with low microbial diversity and reduced *Firmicutes*, has been highlighted in other forms of inflammatory joint conditions, including rheumatoid arthritis, ankylosing spondylitis, and psoriatic arthritis, which are all autoimmune diseases (202, 205, 206).

Dysbiosis has also been observed in other autoimmune conditions, such as insulin-dependent diabetes mellitus (type 1 diabetes), multiple sclerosis, and systemic lupus erythematosus (207, 208). Bacterial translocation, bacterial metabolites, and increased intestinal permeability appear as possible promoters of autoimmunity, highlighting the potential role of the gut microbiota in the pathogenesis of autoimmune diseases (208–210).

Apart from gastrointestinal cancer, other forms of cancer have been associated with gut microbial dysbiosis, including lung cancer and breast cancer (211–214). Patients with lung cancer have shown different gut microbial composition when compared to controls, including higher abundance of *Enterococcus* and lower levels of *Actinobacteria* and *Bifidobacterium*, lower amount of *Firmicutes* and *Proteobacteria*, and more abundant *Bacteroidetes* and *Fusobacteria* (211, 215, 216). Dysbiosis has also been observed in patients with breast cancer, involving a category of estrogen-metabolizing bacteria that can affect estrogen levels and influence the development and prognosis of the breast carcinoma (217, 218).

Skin conditions such as psoriasis, atopic dermatitis, and acne have also been linked to gut dysbiosis. For example, patients with psoriasis have displayed gut microbiome profiles that resemble the gut changes observed in IBD patients. Atopic dermatitis patients have exhibited low levels of *Akkermansia*, *Bacteroidetes*, and *Bifidobacterium*, while individuals with acne have shown decreased *Firmicutes* and increased *Bacteroides* (180, 219).

Respiratory conditions have also been associated with intestinal bacteria. Atopic asthma, for instance, has exhibited depletion of *Faecalibacterium, Akkermansia,* and *Lachnospira*, and has been associated with the gut colonization during infancy, influenced, for example, by birth delivery route and environmental exposure (13).

Neurological disorders have also been linked to gut microbial dysbiosis, with the gut-brain axis communication system likely to play a role (220, 221). Gut microbial dysbiosis has been shown in, among others, autism, Alzheimer’s, Parkinson’s, Attention Deficit Hyperactivity Disorder (ADHD), and schizophrenia (5, 138, 222). Autism spectrum disorder has been associated with increased *Firmicutes*/*Bacteroidetes* ratio and elevated levels of *Escherichia*/*Shigella* and the yeast *Candida*, while Alzheimer’s disease has been connected to increased *Escherichia*/*Shigella,* increased *Bacteroidetes*, and reduced *Firmicutes*, *Bifidobacterium*, and *E. rectale* (5, 14). Parkinson’s has been related to a reduction in *Prevotella* and an increase in *Bifidobacterium* and *Lactobacillus*, which are associated with reduction in ghrelin, a gut hormone involved in neuronal functions that has low concentration in all patients with Parkinson’s (223). ADHD, the most common neurodevelopmental disorder in young people, is mainly characterized by inattention, hyperactivity, and impulsivity, with gastrointestinal symptoms also appearing as a common characteristic among the patients (224). Multiple studies have investigated the relation between gut microbial profiles and ADHD, showing, for example, low abundance of *Faecalibacterium* and association with *Neisseria* and with *Desulfovibrio*, and sometimes showing or not showing significant differences in microbial diversity indices compared to controls. The heterogeneity of the results could be explained by the limitations that characterize the different study designs, e.g., use of medications, small sample size, and participants selection criteria. These limitations therefore underline the necessity of following standardized methods during investigations (225, 226). The role of the gut microbiota has also been extensively studied in schizophrenia, a chronic, complex psychiatric disorder (227). Neurotransmitters dysregulation, alterations of the immune system functionalities, and irregular neurodevelopment have all been associated with the pathogenesis of schizophrenia. Because of the important role played by the gut microbiota in modulating these neurological and immunological factors, the intestinal bacterial are expected to have an impact in the pathogenesis of this psychiatric condition (228). Patients affected by schizophrenia have also shown higher abundance of *Actinobacteria* and lower of *Firmicutes* compared to healthy controls, while the severity of symptoms have been associated with *Succinvibrio* and *Corynebacterium* abundance (229).

## 5. Health improvement through gut microbiota

**Figure 1:**
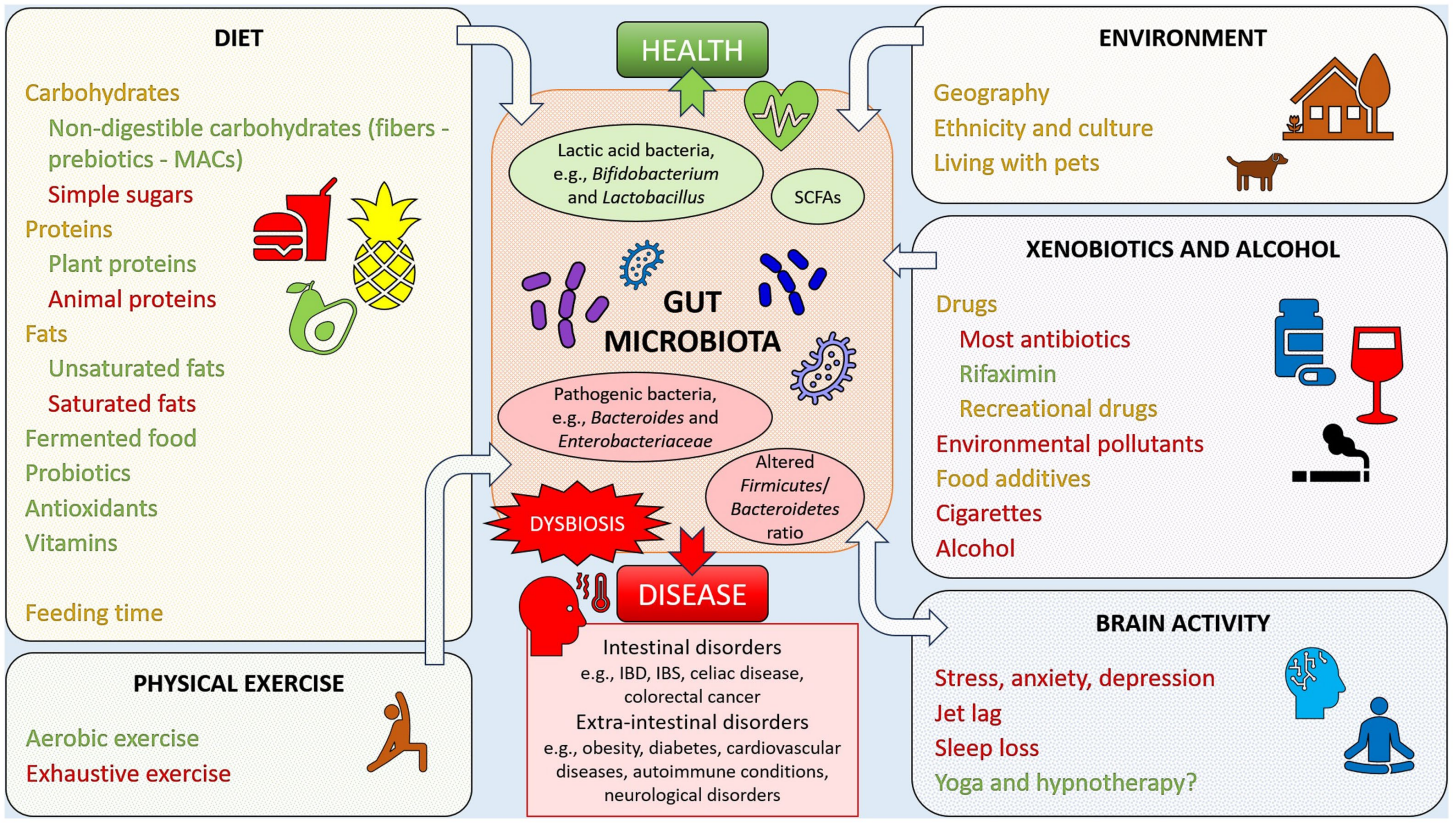
Example of lifestyle factors impacting the gut microbiota, favoring health or disease. Factors reported in green have been linked to positive, health-oriented changes, while factors reported in red have been associated with negative alterations of the gut microbiota, linked with dysbiosis and disease states. Elements reported in orange font are factors with an impact that may be either positive or negative. Yoga and hypnotherapy have been marked with a question mark since it is still not clear if the positive health effects may be linked to gut microbiota changes.

The advantage offered by the link found between health and disease and gut microbial composition is that, targeting the intestinal microbiota, for example by following specific diets and/or taking appropriate supplements, could be employed as a preventive measure or therapy. A summary of lifestyle factors that may impact the gut microbiota, and, consequently, the state of health or disease, is given in Figure 1.

When in abundance in the diet, plant proteins, for example, have been shown to induce increased levels of *Bifidobacterium* and *Lactobacillus*, reduced number of pathogenic species, raised amount of SCFAs, and therefore increased intestinal health. Conversely, animal proteins have been associated with an increase in bile-tolerant anaerobic bacteria such as *Bacteroides*, *Alistipes*, *Bilophila*, and *Ruminococcus*, and a reduction in *Bifidobacterium* and SCFAs, and therefore increased risk of gastrointestinal diseases (182, 230).

High fat diets have been linked to an increase in *Bacteroides* and decrease in lactic acid bacteria such as *Lactobacillus* and *Streptococcus*. However, differences have been observed between diets rich in unsaturated rather than saturated fats, with unsaturated fats inducing an increase in lactic acid bacteria and *Bifidobacterium* and a reduction in blood cholesterol levels, and saturated fats increasing *Bacteroides* and *Bilophila*, lowering *Bifidobacterium* and *Lactobacillus*, and promoting an increase in cholesterol levels and a reduction in insulin sensitivity (182, 230, 231).

Digestible carbohydrates (e.g., sugar and starch) have been linked to increased relative abundance of *Bifidobacteria* and reduced *Bacteroides*, while non-digestible carbohydrates (fibers, referred to as prebiotics, sources of MACs) have been shown to promote bacterial abundance, with an increase in *Bifidobacteria* and *Lactobacilli* and higher SCFAs levels, benefitting the host organism health (14, 182). A diet rich in fiber and butyrate resulting from bacterial fermentation has also been associated with improved brain health (232).

Probiotics have displayed a beneficial role in intestinal health, improving symptoms of, for example, IBD, IBS, gastroenteritis, obesity, cardiovascular diseases, arthritis, and furthermore, stress, anxiety, and depression (14, 43, 137, 191, 198, 233). Supplementation with the probiotic *Akkermansia muciniphila* has also been employed as treatment for obesity, where the beneficial effect can be explained through modulation of insulin sensitivity and glucose metabolism (234). Furthermore, probiotics such as *Lactobacillus rhamnosus* are more frequently being employed in anti-cancer immunotherapy to ameliorate dysbiosis, reduce inflammation, and improve host immunity (235, 236).

Fermented food such as kefir, a source of probiotics, has shown benefits in cases of lactose malabsorption and in the eradication of *Helicobacter pylori* infection (237–239). However, human clinical trials focusing on the health effects of other fermented foods, such as kombucha tea, fermented soy and vegetable products, and sourdough bread are lacking and should be explored to either confirm or dismiss the promising health effects showed by *in vitro* and animal studies (237).

The xenobiotic rifaximin has also emerged as an effective method to reduce inflammation and improve IBD and diverticulitis symptoms (240).

Several vitamins have been shown to increase the abundance of certain gut microbial commensals, including SCFAs-producing bacteria. By promoting bacterial diversity and richness, and increasing SCFAs production, health is also promoted (231, 241). For example, antioxidants such as polyphenols, vitamin B2, and vitamin C have shown potential in improving dysbiosis when increased in the diet (137, 182). Polyphenols found in tea, coffee, red wine, berries, and vegetables have been shown to promote the abundance of butyrate producers, *Bifidobacteria, Lactobacillus*, *Bacteroides vulgatus*, and *Akkermansia muciniphila*, and to reduce the numbers of lipopolysaccharide producers (199, 242, 243). High daily consumption of coffee, which is rich in polyphenols, has been associated with higher abundance of *Bacteroides*–*Prevotella*–*Porphyromonas,* which could be useful in the treatment of conditions where *Bacteroides* species are generally depleted, such as obesity (244). In another study, coffee consumption led to an increase in *Bifidobacterium* spp. after 3 weeks with a daily dose of 3 cups, indicating favorable effects on host health (245). Regardless of these findings, coffee consumption should take into consideration individual variability in response to caffeine, as some people may experience increased sensitivity and lower quality of sleep (246). Certain individuals have also appeared to be more sensitive to the effect of coffee on colonic motility (247, 248). Furthermore, coffee consumption has been associated with higher probability of IBS and worse IBS symptoms in certain groups of people (249).

The Mediterranean diet, in general, high in fibers, antioxidants, and unsaturated fats and low in red meat, has been associated with increased lactic acid bacteria in the gut, lower cardiovascular risks, lower obesity, and increased protection against cancer. On the contrary, the Western diet, high in animal fats and proteins, has been linked to higher levels of *Bacteroides* and *Enterobacteria*, higher inflammation levels, and increased health problems (44, 182, 250).

The vegetarian diet has been proven beneficial to the immune system and capable of inducing a change in the gut microbiome composition, while the vegan diet, rich in dietary fibers and antioxidant, has been associated with health benefits in relation to medical disorders such as diabetes and hypertension. However, strict diets may also pose a health risk if not well planned, as individuals may suffer from nutritional deficiencies (251, 252).

The ketogenic diet, exceptionally low in carbohydrates and high in fat, characterized by the induction of low insulin levels and a state of ketosis in the body, has been identified as beneficial for protection against cancer, epilepsy in infants, autism, and multiple sclerosis (44). However, little is known about the effect of the ketogenic diet on the gut microbiome composition, with some studies reporting negative and other positive effects (253).

The low FODMAP (fermentable oligosaccharides, disaccharides, monosaccharides, and polyols) diet, characterized by a reduction in the consumption of food containing short-chain carbohydrates which are poorly absorbed in the gut, has been proven beneficial in reducing IBS symptoms in 50–80% of patients (254). Gut microbiome changes associated with this diet have also been observed, including a reduction in relative abundance of *Bifidobacterium*, likely caused by the decreased intake of prebiotics such as fructans and galacto-oligosaccharides (255, 256). The reduction in prebiotics intake indicates that following a strict low FODMAP diet could be counterproductive, leading to reduced *Bifidobacterium* strains and dysbiosis. However, the clinical significance is currently unknown and should take into consideration the recommendations of following a strict low FODMAP diet for a maximum of 2–6 weeks (257, 258). On the other hand, individuals with IBS microbiota profiles characterized by pathogenic properties (low bacterial diversity, low levels of *Bacteroidetes* commensals, and enriched *Firmicutes*, including pathogens) exhibited a marked change in microbiota composition (increased *Bacteroidetes*/decreased *Firmicutes*) associated with fewer IBS symptoms after 4 weeks of following a low FODMAP diet (259). Further studies are therefore needed to fully understand the impact of the low FODMAP diet on the gut microbiota and maintain control over the drawbacks.

In addition to the specific diet adopted, dietary timing has also displayed the ability to affect the gut microbiota. Intermittent fasting, an eating pattern composed of periods of fasting alternated with period of eating, has been linked to gut microbiota changes in mice and health promoting effects in humans (260–262).

Besides dietary interventions and probiotics and prebiotics supplementations, the more invasive fecal microbial transplantation (FMT) has also been used as a form of therapy directed at the gut microbiota. FMT is a technique that allows a donor to receive gut microbes from a healthy individual and has been proven effective not only to contrast *C. difficile* infection, but also against IBD, metabolic syndrome, and autism (13, 14). However, careful donor screening and caution are required to reduce the probability of side effects, as long-term outcomes are still not fully understood (263).

Given the demonstrated effect on the gut microbiota composition, exercise has also been proposed as a potential therapeutic method for conditions such as IBD. However, the negative effects revealed by exhausting exercises and the positive effects showed by aerobic exercise, especially when adopted for a duration of more than 12 weeks, should be kept in mind (264, 265). IBS also appeared to benefit from increased physical exercise over a period of 12 weeks, which led to an improvement of symptoms in a 2011 randomized controlled trial (266). However, the causes of these benefits may not rely on the gut microbiota composition but more so on improved intestinal motility, reduced blood flow in the gut, or on the general improvement of the quality of life of the study participants performing regular exercise (267).

Along with diet and exercise, the emotional state of individuals, e.g., stress has been shown to affect intestinal conditions such as IBD and IBS, triggering a worsening of the symptoms (268). Methods for managing stress levels including yoga have, in fact, been proven effective in reducing IBS symptoms (269). Hypnotherapy directed to the gut, called gut-directed hypnotherapy (GDH), has also been gaining popularity as a treatment for IBS symptoms, showing efficacy comparable to the adoption of a low FODMAP diet (270). As a result of the success obtained with GDH, the American College of Gastroenterology has added this practice among the clinical guidelines for IBS management (271). However, the involvement of gut microbiota changes in mediating some of the positive effects of relaxation techniques and GDH has still not been clarified. The first study investigating the effect of GDH on the gut microbial composition of patients with IBS showed only small microbial changes, while the symptoms of IBS reduced significantly, indicating that GDH-mediated effects might be independent from the microbiota composition (272).

## 6. Personal genomics

### 6.1. Popular investigative methods

The analysis of microbiota composition has evolved from lengthy culture-based method to high-throughput next-generation sequencing techniques that enable a relatively fast analysis of the genomic content of the microbes contained in stool samples (273). To obtain consistent and comparable results, care should be taken in deciding what processing method to use as bias could be introduced at various stages, starting from the choice of collection method and storage conditions (274). If fresh fecal collection is chosen, it is generally recommended to promptly transfer the collected specimen to the laboratory on dry ice within 24 h of collection (275). This is to limit bacterial growth between collection and processing, as bacterial taxa differences have been observed as little as 15–30 min after stool deposition at room temperature. Nevertheless, other sources showed no significant changes in sample composition within 24–48 h from collection at room temperature (276–278). The current gold standard for fecal storage consists in freezing the samples at −80°C as soon as possible after collection, but this represents an obvious challenge for participants collecting their samples at home (279). To ease collection, preservation buffers are also available, including the popular, user-friendly OMNIgene GUT (DNA Genotek). Collection with this kit has showed good comparability with both fresh and freshly frozen samples, with some data also suggesting greater DNA yield and quality compared to fresh samples, due to improved DNA shearing (280–284). During sample collection it is also important to consider the uneven distribution of bacteria on a deposited fecal sample, which requires the definition of a standardized approach. This might include homogenization or mixing of the sample prior to processing (277, 285–287).

When estimating the effect of sample storage, extraction method, biological variability, and sequencing method, it has been shown that the DNA extraction process may have the biggest impact on the results (274). In a 2019 study comparing 6 extraction methods, different extraction techniques resulted in not only dissimilar DNA yield, but also differing microbial profiles with significant variation in *Firmicutes*, *Bacteroidetes*, and *Actinobacteria* distribution (279). Furthermore, some extraction protocols have demonstrated higher efficacy at retrieving genomic content from thick-walled gram-positive bacteria compared to others (288). On this regard, the introduction of a mechanical disruption process through bead beating has been proven beneficial to increase microbial cell lysis and improve DNA recovery, and it is therefore often recommended (288, 289). In an attempt for standardization, the International Human Microbiota Consortium (IHMC) started the International Human Microbiota Standards (IHMS) project comparing 21 DNA extraction protocols for human stool. Out of these, the best performing in terms of quality, transferability, and reproducibility was Protocol Q (274, 290, 291). This protocol was also assessed along with other 4 methods in a 2019 study and it appeared to perform better than the other procedures (292). Likewise, another study showed increased performance of this protocol compared to QIAamp PowerFecal Pro DNA Isolation kit (Qiagen) (293). On the other hand, when compared to a simple and economic method called MetaHIT, Protocol Q showed lower performance (279). In this same study, the choice of extraction method had more impact on the results compared to different short-term sample storage, confirming previous findings (274, 279).

One of the most common methods to define gut microbial composition is the targeted sequencing of the bacterial 16S small subunit ribosomal RNA (rRNA) hypervariable regions, which allows taxonomical classification based on similarities of the targeted region (294, 295).

**Figure 2:**
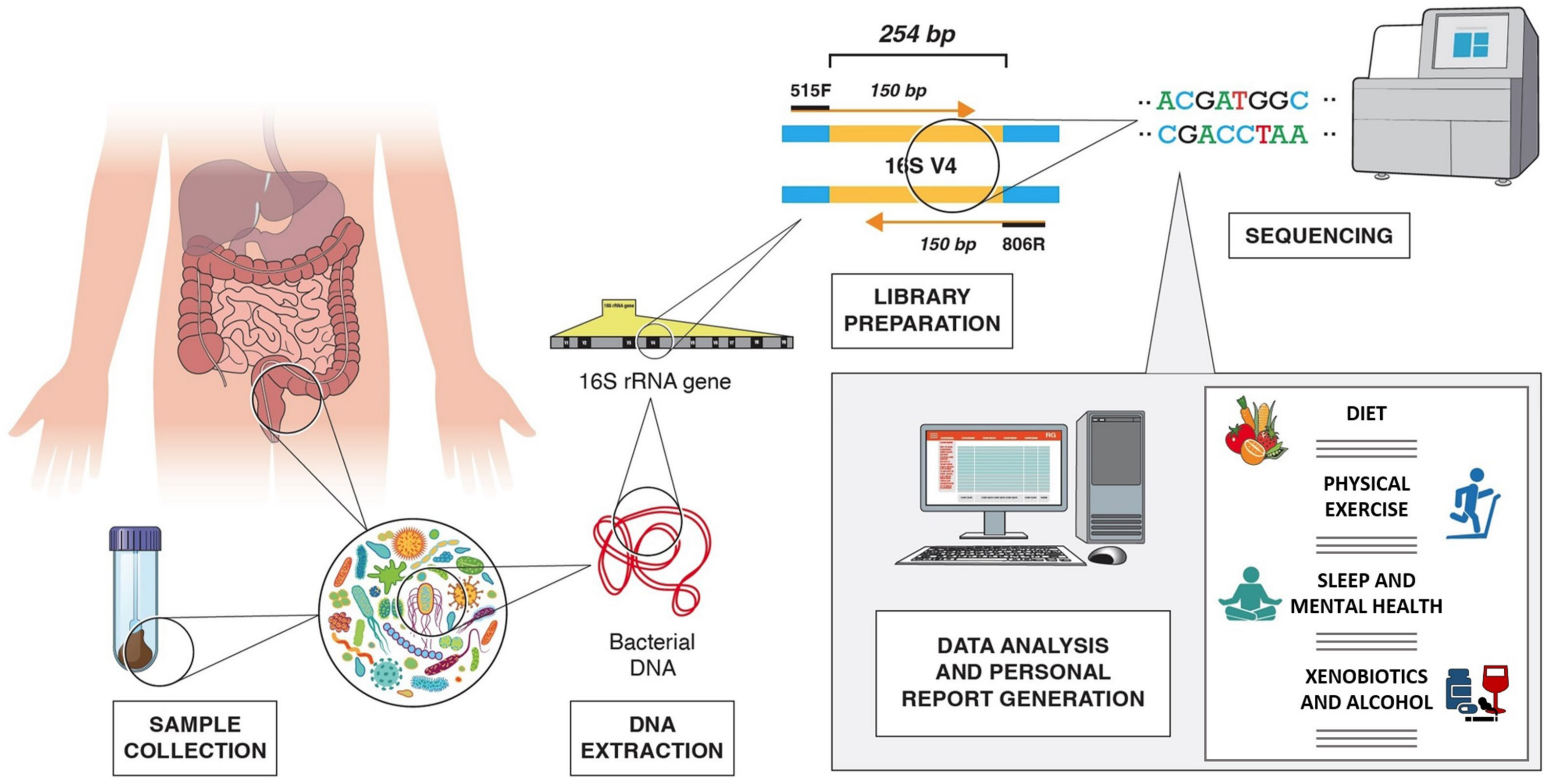
Example of V4 16S rRNA sequencing workflow, from fecal sample collection to generation of personalized lifestyle report, including dietary suggestions, physical exercise, sleep, mental health, xenobiotics, and alcohol use recommendations. In the example, the 254 bp long V4 region is amplified with 515F and 806R primers.

The 16S rRNA genetic sequence, a 1,550 bp-long sequence found in prokaryotes, contains both conserved regions and nine hypervariable regions, V1–V9. These sequences can be targeted for amplification using universal conserved primers to create sequencing libraries. The sequencing results can then be used to identify the microbial composition of the sample through comparative taxonomy (294, 295). Nonetheless, it has been shown that targeting different regions of the 16S rRNA gene can affect the results as different regions provide different discriminatory power for taxonomic classification (296). Most studies on the human gut microbiome have been carried out using V3 or V4, or a combination of two or more hypervariable regions, most commonly the V3/V4. However, the Metagenomics of the Human Intestinal Tract (MetaHIT) consortium and the Earth Microbiome Project have highly recommended V4 as the gold standard (297). The taxonomic discrimination efficacy obtained when targeting different hypervariable regions showed that either paired end V3 or V4 regions, with sequencing length of only 100–120 nucleotides, deliver the best results, even though an underestimation of species richness was reported when using V3 compared to V4 (295, 298). The sequencing platform available should also be taken into consideration as different platforms may allow for different sequencing length. For example, the MiSeq System (Illumina) sequencing platform, a widely used instrument for microbial sequencing, allows for a 300-base pair (bp) x2 read length, providing accurate and efficient characterization of up to 600 nucleotide targets sequence. Other sequencing platforms, however, are limited to 150 bp x2 read lengths (299, 300). Among the different hypervariable regions, V4 has the advantage of being short enough to allow for 150 bp x2 sequencing and create an overlap of around 50 bp (301). For instance, the universal primers 515F and 806R generate a 254 bp-long amplicon in the V4 region, allowing the use of 2×150 bp sequencing [Figure 2; (302)]. These are also suggested by Illumina as the primers to use when employing 2×150 bp sequencing using the MiSeq platform (303).

Targeting bacteria only, rather than including fungi, protozoa, and viruses, can be justified with the fact that more than 90% of the genes obtained with metagenomic approaches are of bacterial origin (17, 25). The 16S rRNA method has the advantage of being cost-effective, fast, and applicable to low-biomass samples contaminated with host DNA. However, it can introduce bias due to variable primer affinity, amplicon size, and number of PCR cycles. In addition, targeting short 16S rRNA regions often leads to lack of discrimination at the species level (275, 304).

Nevertheless, with the use of more recent long-read technologies, full-length 16S rRNA sequencing is also possible, with resolution down to the species level. An example is provided by the MinION Nanopore (Oxford Nanopore Technologies), which records the differences in voltage across an electro-resistant membrane caused by the passage of nucleic acids through nanopores (305, 306). Other advantages of the Nanopore are the cheap cost, the possibility of visualizing results in real-time, the option of processing small sample numbers with contained costs, and the quick library preparation time. However, disadvantages are also present, including the high amount of starting nucleic acid material required (few micrograms of DNA) and the low accuracy compared to Illumina platforms, even if rapidly improving with new versions (307).

In alternative to 16S rRNA sequencing, the whole-genome shotgun metagenomic approach is frequently used. The whole-genome shotgun metagenomic method, which targets all the nucleic acid present in the sample (bacterial, viral, eukaryotic, and host DNA), can provide a more reliable estimate of the microbiome composition as it does not introduce PCR-dependent bias and it can discriminate down to the species and even strain level. Given the genes relative abundance, it can also provide potential functional information (275, 299, 304, 308). However, shotgun sequencing is more expensive and time consuming, which represents a limitation for commercial use or clinical testing (275, 308). Also, the use of different whole genome sequencing protocols, instruments, and bioinformatic analysis has been associated with different relative abundance of species for the same samples, introducing bias (309).

A limitation of these metagenomic approaches compared to culture-based method is the potential loss of information on low-abundance strains as well as low strain-resolution accuracy, problems that can be addressed by combining metagenome-assembled regions with newly discovered single-cell metagenomic methods. However, these methods remain prohibitive in terms of costs and analysis challenges (310, 311). New microfluidic methods based on droplets containing microbial cells from fecal samples are also under evaluation to produce complete and precise microbial characterization that would enable an improved definition of overall microbial function (311, 312).

A step further is represented by the metatranscriptomic approach, which provides information relative to only actively expressed genes and therefore distinguishes between dead and alive organisms. In spite of this, it can be a difficult method from both a technical and analytical point of view, and it is not cost-effective (275, 308).

Metaproteomic and metabolomic analyses, which are commonly based on Mass Spectrometry analyses, can also be employed. These techniques allow for the definition of the gut microbiome sample functional profile and metabolic activity and can be used in combination with bacterial taxonomic identification to better understand the role of bacteria in the gut (313).

The direct analysis of stool consistency and pH has also been shown to offer an indication of the gut microbiome and metabolic composition.

**Figure 3:**
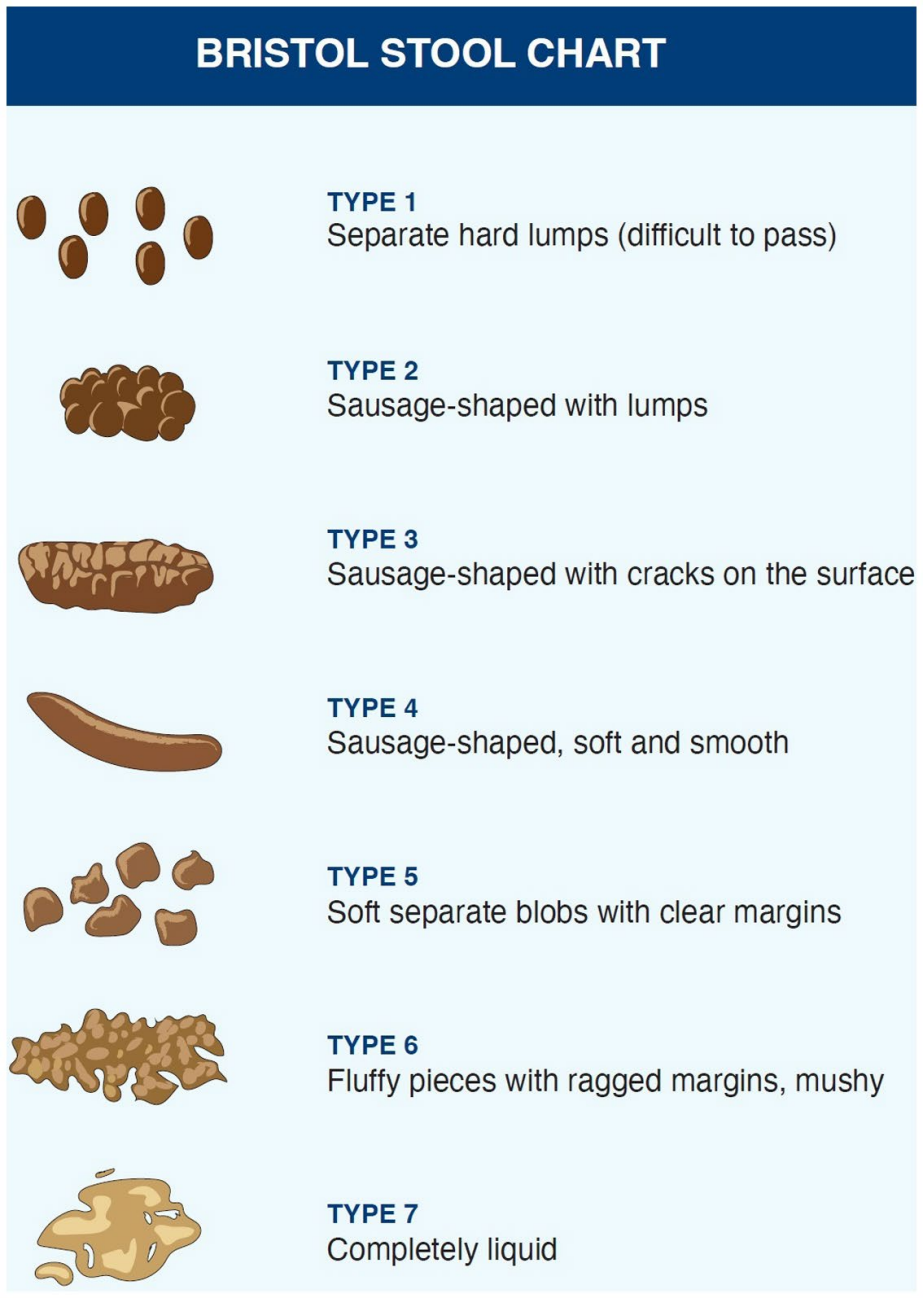
Feces types on the Bristol Stool Chart.

The Bristol Stool Chart (BSC), a scale of stool density which is widely used as a method to assess intestinal transient time, consists of a 1 to 7 scale, from very hard stool to gradually increased water content [Figure 3; (314, 315)]. It has been shown that the higher the BSC score is, the lower the bacterial richness and the higher the *Bacteroidetes*:*Firmicutes* ratio are. *Prevotella* enterotype was found to be more abundant in individuals with higher BSC score, while *Ruminococcaceae*-*Bacteroides* (RB) enterotype was more commonly associated with lower BSC score. Within the RB enterotype, *Methanobrevibacter* and *Akkermansia* appeared higher with longer colon transit time, while microbiota growth potential (the ability of bacteria to grow fast) was found to be higher with higher BSC scores (316). The degree of variability determined by the BSC has also been identified as a potential, important confounding factor in the obtained gut microbiota composition. In fact, the latter is affected by the ability of different bacterial species to grow quickly in unstable environments, e.g., diarrhea and is also affected by the time available to adhere to the intestinal mucosal layer (317, 318).

The stool pH has also been identified as an indicator of intestinal parameters such as dietary fiber intake and butyrate levels. In the gut, bacterial fermentation converts soluble fibers into SCFAs, which are weak acids, causing a reduction in stool pH and therefore leading to a negative relationship between butyrate and pH (319, 320). The increased intestinal transit rate caused by dietary fibers could also lower the stool pH as a result of the reduced time available for absorption and consequent elevated SCFAs concentration in the stool (321). Fiber-rich vegan diet, for example, showed an association with fecal pH of ~6.4 compared to ~6.7 of omnivore diet (322). It is also believed that high pH may be associated with higher risk of colonic cancer, while people at lower risk consuming high amount of fiber produce more acid and solid stool (323). Low pH may also affect bacterial growth and increase the levels of SCFAs-producing bacteria. Given the important role of SCFAs in gut health and their effect on the gut pH, lower pH values could therefore be associated with better intestinal health (321, 322).

### 6.2. Personalized healthcare

A wide variability of gut microbial composition can be observed in the population, depending on genetics, but also, lifestyle choices, age, diseases, medications, and other factors. On top of this, the role of intestinal bacteria in defining the overall health state of an individual appears crucial. As a result, the opportunity to characterize someone’s profile to provide personalized lifestyle indications has arisen (15, 261, 318, 324, 325).

More and more laboratories worldwide are starting to offer gut microbiome testing to provide, for example, health scores of microbial composition, nutritional advice, and supplementation suggestions. Personalized nutrition, in particular, aims at applying specific diet changes to individuals with known microbial composition in order to trigger the desired change, with health in mind (261). However, potential limitations in methodologies and current knowledge should be taken into consideration to evaluate the significance of such testing. As discussed above, from a methodological point of view, bias could be introduced during testing at several stages. Collection method, storage conditions, extraction method, sequencing protocol, data processing, and analysis are all variables that can affect the analysis of gut microbiota composition from fecal samples. Additionally, the lack of use of standardized methods leads to reduced comparability of the results and reduced confidence over the meaning of the observed variability. The lack of methodological consistency in gut microbiome studies therefore represents a significant limitation, generating possible bias and affecting reproducibility (261, 318). Methods should be chosen carefully and consistently, taking into consideration feasibility, cost, time, and accuracy. In addition, attempts to define a gold standard workflow for gut microbiome testing should proceed and be adopted.

Another prime aspect to consider for the delivery of personalized advice is how microbial variability is defined and what elements should be considered to produce such advice, i.e., *Firmicutes/Bacteroidetes* ratio, presence/absence of specific bacteria. The adoption of enterotypes, for example, has helped in defining differences in microbial composition (17). However, especially when considering the gut microbiota of healthy individuals, there is still a high degree of variability due to genetic and environmental factors that is yet to be fully explained (318).

An aspect that should not be overlooked in personalized nutrition is represented by the emerging realization that the response to dietary intervention is variable between individuals and may be affected by factors such as host physiology, age, gender, dietary habits, and baseline microbiota composition. In this regard, initial microbial diversity, microbial stability, and gene richness could all affect the outcome of planned interventions (15, 326). For example, individuals with enterotype characterized by high *Prevotella* to *Bacteroides* (P/B) ratio appeared more prone to body fat loss when consuming a high fiber diet for 6 months compared to individual with low P/B ratio (327). Similarly, improved glucose metabolism was observed in individuals with a higher baseline P/B ratio after 3 days of a diet rich in barley kernel-based bread (328). In another example, the presence of *Eubacterium ruminantium* and *Clostridium felsineum* correlated with increased health benefits in individuals with obesity subjected to simple dietary changes (329). The success of a low FODMAP diet administered to individual affected by IBS may also be influenced by the initial gut microbial composition, even if the achievement of a prediction method is limited by differences in the study designs that can affect the results (330). The different interindividual response to interventions could be due to the different ability of bacteria to metabolize the food present in the provided diet. As bacteria compete for nutrients, specific interactions between microbial species may also have an effect. Additionally, it has been shown that bacterial species could be depleted and could face extinction after prolonged unhealthy diets, leading to requirements for probiotic and prebiotic interventions coupled with healthy diets to increase the chances of observing positive outcomes (15, 331). However, there is still lack of knowledge in the variables that could impact the response to dietary changes and more research is required to evaluate each factor and maximize the results of interventions (326).

In order to evaluate the specific response of an individual gut microbiota to different dietary components, gut-on-a-chip bioreactor system technology could also be employed. The response, evaluated as changes in SCFAs production, would allow for personalized prescription of food type and quantity that leads to higher SCFAs levels. However, this complex technology is still recent and requires further research to overcome the current limitations (332, 333).

In an attempt to predict how the human gut microbiota composition influences the response to dietary, prebiotic, and probiotic interventions, *in silico* approaches are starting to be employed, including artificial intelligence, statistical modeling, and mechanistic modeling. Given the possibility of putting together gut microbiota data with clinical information, genetic information, blood parameters, dietary habits, and any other useful data about an individual, *in silico* approaches employing artificial intelligence are emerging as the future of microbiome-based personalized healthcare (332). With the aim to improve the power of personalized nutrition, the National Institutes of Health (NIH) has started Nutrition for Precision Health, powered by the All of Us Research Program, to build algorithms able to predict the individual response to dietary interventions (334). The European Union-funded PROTEIN project (PeRsOnalised nutriTion for hEalthy living) also aims to develop a mobile phone application that utilizes user data, such as health status, eating behavior, physical exercise, physiological parameters, genetic and gut microbiota data to deliver real-time personalized dietary and physical activity advice (335). The concept includes the use of sensors to monitor physical activity, bowel movements, food intake, glucose levels, and volatile organic compounds, the latter contributing to define the gut microbiota composition (335, 336). The PROTEIN project emerges as an effort from experts in the field to provide advice based on sound science, in a world were m-Health (mobile health) applications providing nutritional and fitness guidance are increasing in popularity (335). The Stance4Health (Smart Technologies for personAlised Nutrition and Consumer Engagement) Nutritional APP is also a project funded by the European Union, through the Horizon 2020 research and innovation program, with the objective to develop mobile technologies for personalized nutrition (337, 338). The aim of the APP is to generate menus tailored to the user’s characteristics, including a gut microbiota module based on 16S rRNA sequencing from fecal samples. A preliminary study showed promising results, providing nutritional education and useful interventions advice that could have a positive impact if used for a prolonged period of time (337). The mobile application Zoe has also recently emerged and gained popularity, providing personalized nutritional advice based on blood fat and sugar, and gut microbiome testing (339). As a collaboration of scientists from renowned institutions (Massachusetts General Hospital, Stanford Medicine, Harvard T.H. Chan School of Public Health, and King’s College London), Zoe has published numerous studies, called the Personalised Responses to Dietary Composition Trial (PREDICT) studies, in support of the testing and advice offered (340). These examples represent efforts to generate mobile applications and softwares backed by science amid the multitude of mobile applications that are emerging, some of which might not be as attentive to proper evaluation. Health-related apps have become increasingly popular, but the majority of them still appear to be made available to the public without the necessary preliminary scientific evaluation (341).

Personalized diet could therefore be provided in two ways: by identifying specific relationships between the gut microbial profile and host metabolic properties, e.g., presence/absence of specific bacteria or genes that could be introduced by adapting the diet; by creating specific algorithms; by using machine learning and artificial intelligence to combine multiple host data. The latter is a very powerful method as it overcomes the lack of knowledge on the underlying mechanisms by taking into account variables that otherwise would be difficult to capture. However, not understanding the reasons behind the delivery of a specific diet can be seen as a disadvantage, and it underlines the importance of using an integrated approach to advance in the field (261). Successful prediction of the glycemic response after meals has been achieved by using machine learning to combine blood parameters, gut microbial information, dietary habits, physical activity, and anthropometrical data, followed by personalized nutritional interventions to lower the postprandial blood sugar level (342). Interestingly, the same study showed that the exclusion of data such as blood tests and medical questionnaires results in only a small loss of accuracy in prediction, highlighting the relevance of gut microbiome information and the possibility of performing gut microbiome analysis only to provide sufficient nutritional advice (342, 343).

The plasticity of the gut microbiota and related analysis would also allow for follow up and repeat testing, enabling the evaluation of the effect of the adopted interventions, which can cause changes in the gut microbiota community that might require reassessment and readjustment until an equilibrium is reached (343).

In the context of personalized advice, it is imperative to integrate microbiome information with individual human genome characteristics, which determine the host’s nutrient metabolism. In this regard, nutritional genomic studies provide insights into the genetic profile associated with, for example, lactose intolerance, caffeine sensitivity, and vitamin metabolism. Dietary advice modeled on genetic differences therefore allows for a more complete definition of each individual’s nutritional requirements and their reaction to certain dietary elements (344–346). Integration of genomic results with gut related proteomic and metabolomic information could also be used to provide additional information on gut microbial functions (313).

When it comes to exercise, there is still a lack of resources combining the gut microbiome to specific personalized advice. However, human genetic markers have already been used to provide personalized recommendation in terms of endurance ability, muscle performance, injury recovery, and motivation to exercise (347). Further studies in the relationship between gut microbiota and fitness should be undertaken to eventually be able to combine genetic factors and microbial data to provide accurate personalized advice.

Because of the scarcity of data available on the bidirectional influence between gut microbiota and emotional states in humans, more research is still required to fortify the identified possible links and be able to include specific stress management activities as promoter of healthy gut microbiome composition.

### 6.3. Ethical considerations

While personal genomics and microbiome research hold immense promise to advance our knowledge of health and disease, they also give rise to significant ethical and privacy considerations that demand careful attention. These concerns span multiple domains. Ethically, participants must be granted comprehensive informed consent, understanding both the research and testing nature and potential risks and benefits, alongside their right to withdraw and knowledge about data utilization and sharing. Particular attention should also be given to the type of data that might be obtained through such testing, which should guarantee the expectations of the users are not betrayed. If an individual is signing up for personalized lifestyle advice, for example, they might not want to receive any clue on the presence of genetic or pathogenic conditions that might impact their overall health. In the case these are collaterally identified by the laboratory, procedures should be in place on how to treat this type of information, according to what was initially agreed with the client and the type of informed consent that was signed for (348–350).

The principles of privacy and confidentiality are paramount, necessitating rigorous data protection measures such as de-identification or anonymization. Researchers also bear the responsibility of ensuring fairness and equity in the distribution of research benefits and risks, particularly across diverse populations, while avoiding the exploitation or exclusion of vulnerable groups. Moreover, the delicate balance between data sharing advantages and re-identification risks calls for ethical and responsible practices. Privacy concerns emerge in the form of sensitive genetic and microbiome information, affecting health, employment, and insurance, necessitating participant awareness and control over their data. Furthermore, safeguarding against data breaches, given the attractiveness of such information to malicious actors, is of utmost importance (351, 352).

In essence, as personal genomic and microbiome research and testing advance, researchers must forge close partnerships with participants, regulators, and stakeholders to conduct ethical research that upholds the rights and dignity of all involved parties. Private testing companies must keep in mind users’ rights and have policies in place focused on avoiding any type of harm or unnecessary distress to the people involved in the testing.

## 7. Conclusion

The recent advantages offered by next-generation sequencing methods have opened the way to relatively fast and detailed investigations of the gut microbiota composition, leading to numerous studies focused on the associations between the gut microbiome and human health. It soon appeared that human guts are mainly inhabited by *Bacteroidetes*, *Firmicutes*, *Actinobacteria*, *Proteobacteria*, *Fusobacteria*, and *Verrucomicrobia* phyla, but that wide variation in composition and relative abundance is present among different individuals. Further analyses suggested the possibility of grouping different profiles in 3 clusters of variation, enterotype I (rich in *Bacteroides*), II (rich in *Prevotella*) and III (rich in *Ruminococcus*), but more studies highlighted the limitation of such system and introduced more complex classifiers. The difficulties encountered in gut microbial profiles classification is relevant in the context of diagnosis and development of personalized lifestyle advice, since defining gut microbial variability and establishing discrete groups facilitate the discovery of association. Hence, more efforts are needed to define the spectrum of gut microbiome healthy and diseased signature states and achieve standardization during study design.

Significant differences have been observed among different populations observing different diets, shaped by the relative intake of fiber, fat, and proteins. Fiber-rich diets have been associated with high abundance of *Prevotella* strains, increased gut microbiome diversity, and increased production of SCFAs, butyrate in particular, promoter of intestinal health. Saturated fat and animal protein rich diets have been associated, on the contrary, with an abundance of *Bacteroides* and reduced gut health. The realm of macro and micronutrients, however, is ample and there is further need to evaluate potential association with any type of food to increase the knowledge on their effect on the gut microbial structure. The effect of geography, environment, and ethnicity should also be taken into consideration as their influence on the gut microbiota might not be negligible and could affect studies focused on investigating other parameters. The use of medications and the exposure to heavy metals and other xenobiotics may also exert a negative effect on the human gut microbiota, but further research is required to fully understand the type and dosage of substances that would significantly affect the gut bacteria. All these factors, diet in particular, lead to not only differences between people, but also differences within the gut microbiota of the same person in different moments of their lives.

Altered gut microbial composition, i.e., dysbiosis, has been associated with several conditions and diseases, from intestinal related to neurological. Generally, the gut microbial profile of individuals affected by intestinal disorders and by several other extra-intestinal conditions has exhibited low bacterial diversity, an altered number of lactic acid and SCFAs-producing bacteria, and an altered *Firmicutes* to *Bacteroidetes* ratio. The direction of shift of this ratio appears to relate to certain conditions rather than others: for example, obesity and IBS are often characterized by an increased ratio, with more *Firmicutes* and less *Bacteroidetes*, while conditions such as IBD and type 2 diabetes tend to correlate with less *Firmicutes* and more *Bacteroidetes*. This indicates how increasing the knowledge on diseases and their gut signature profiles can provide fundamental information on gut-directed treatments. In fact, a specific mixture of probiotics and specific diets could be designed to restore balance specifically to the desired effect, as they might promote and hinder the growth of certain bacterial strains rather than others. However, contrasting results have also been identified, with the same conditions being associated to a reduced or increased ratio in some studies rather than others, increasing the need for individual testing and personalized interventions.

In general, increasing the levels of *Bifidobacterium* and *Lactobacillus* through a diet rich in plant proteins, unsaturated fats, non-digestible carbohydrates, and with probiotic supplementations has been proven beneficial for intestinal health. Several special diets could also promote health, but caution should be applied when adopting strict diets such as vegan or low FODMAP, especially when followed for an extended period of time, as negative collateral effect could undermine the positive outcomes.

Exercise has also emerged as a contributor to gut microbiome variation, with amateur aerobic exercise yielding improvements such as increased gut microbial diversity and an increase in SCFAs-producing bacteria. Exhaustive exercise performed by athletes, on the other hand, has been linked to negative effects on gut health. However, difficulties in distinguishing between the effect of exercise and diet in athletic individuals has represented a limitation for several studies. More research is therefore needed to standardize studies and to define the type, frequency, and duration of exercise associated with benefits rather than detrimental health outcomes.

The emotional state of individuals, including mood, stress, and anxiety, has also appeared to influence the gut microbiota composition, and vice versa. Multiple animal studies have investigated the potential mechanisms behind this correlation, while human studies show significant variations and positive effect of probiotics. Even if specific associations between bacterial species and mental health conditions are still not clear, the link with dysbiosis in humans is strong and calls for increased attention toward the gut microbiota and lifestyle choices that can improve mental health. The bidirectional link found between circadian rhythm and gut bacteria also reinforces the need for careful choices not only on the food type, but also on the eating timing, and highlights the impact of jet lag, insufficient and disturbed sleep, on the gut balance. The impact of mental health and sleep disturbances on the gut and the role of the intestinal microbiota in regulating overall health also indicates that by controlling them, general health could improve. Stress management methods such as yoga and hypnotherapy have been employed to contain the symptoms of, for example, IBS. However, the link with changes in the composition of the microbiota in the gut is still unclear.

The possibility of detecting individuals’ gut microbiota composition coupled with knowledge around the impact of lifestyle on the gut microbiome opens the way to personal genomics and personalized lifestyle advice aimed at improving gut, and therefore overall, health. However, the challenges and limitations surrounding this field are still abundant. All the variability in gut microbial composition between and within individuals, and the variable effect observed with certain interventions according to the baseline gut microbiota of the people involved, generate a layer of complications to personalized testing that needs to be investigated and better understood to generate useful advice.

From a methodological point of view, it is important to know what techniques are available and the possible bias introduced with different approaches. The lack of adoption of standardized methods leads to loss of control over variability and comparability of results and encourages the gut microbiome research community to adopt consistent gold standards to improve reliability. Pros and cons of each method should be considered to establish what is the best choice with the resources available (time and finances) and for the result desired (e.g., genera vs. species-level identification, bacteria vs. all microorganisms, dead or alive organisms, etc.). Quick checks as stool consistency and pH level could also be evaluated to suggest enterotype and SCFAs abundance, indicating if more fibers are needed in the diet.

Apart from the processing methods used, the analysis of the results represents an additional challenge. Nutritional advice could be defined on the basis of gut microbial taxonomic information only or by taking into account interpersonal variability, the effect of diet, medications, exercise, and other personal parameters to seek the production of a more complete report. More research is needed to define the more suitable approach and to progress in the definition of the interactions between lifestyle and gut microbiome. Furthermore, any inference made should be based on sound scientific research and caution should be employed when considering interactions identified on animal studies only.

In the technological world we live in, mobile phones can be used as a tool to monitor our health and provide advice in real-time. Studies involving the use of such applications, coupled with user’s information, including eating habits, health conditions, blood parameters, genetic and gut microbiota testing, as well as adding the use of sensors, have shown some promising results, allowing the real-time adaptation of recommendations and providing nutritional education. With this purpose, many companies have been creating their own algorithms to provide nutritional and lifestyle advice in the form of user-friendly applications. Even if these tools have shown potential to improve people’s lifestyle and health, the reliability of some of these applications is questionable as they may not be based on sound scientific research. Even when based on scientific data, the plethora of factors influencing the gut microbiota composition represents a challenge, complicated by the presence of occasional contradicting results. These could be due to study design, methodological, or biological differences, among other confounding factors. On top of these inherent complications, personalized targets might be difficult to achieve, not only because of individuals’ biological differences, but for psychological factors that may also come into play. In fact, an individual’s adherence to the provided advice might not always be thorough, potentially affecting the desired outcome. Because of all these potential issues, it is even more fundamental that softwares and algorithms for personalized nutritional advice are thoroughly validated before use.

The other aspect to keep in mind with the advancement of technological approaches is the use of the generated data and the possibility of data leaks. Personal genomics and microbiome research offer significant potential for advancing our understanding of health and disease. However, they also raise important ethical and privacy issues that require careful consideration. As personal genomics and microbiome research progresses, researchers must collaborate closely with participants, regulators, and stakeholders to conduct ethical research that respects the rights and dignity of all involved parties.

The future direction of gut microbiome testing is a dynamic realm of active research and development, encompassing several promising avenues. Precision medicine stands at the forefront, as scientists strive to unearth biomarkers capable of tailoring medical interventions according to an individual’s gut microbiome composition, potentially revolutionizing treatment strategies for diverse conditions. Concurrently, the integration of microbiome-based therapeutics has solidified its position within modern medicine, necessitating a rigorous assessment of current capabilities and the identification of fundamental research areas to propel future advancements. Metagenomic profiling, driven by advances in sequencing technologies, has enhanced our understanding of microbial communities and their associations with disease phenotypes, as cost-effective profiling becomes increasingly accessible, paving the way for wider application. Envisioned on the horizon is the integration of molecular analysis of microbiomes into clinical settings, promising enhanced diagnostic precision and personalized treatment. Additionally, ongoing research is paving the path for the development of potential screening tools, enabling clinicians to gather pertinent information before embarking on comprehensive gut microbiome sequencing. In essence, the future of gut microbiome testing is highly promising, poised to impact not only the world of personalized lifestyle, but also precision medicine, microbiome-based therapeutics, and more, offering a beacon of hope as innovative approaches continue to evolve in our quest to comprehend and harness the potential of the gut microbiome.

In short, there is an extensive amount of research correlating lifestyle factors with gut microbial composition and general health. However, methodological standardization and more studies focused on identifying causal relationships between lifestyle choices and gut microbiota changes should be undertaken to be able to employ the power of the gut microbiome to improve health and lifestyle. Caution should be exercised when relying on gut microbiome testing services and lifestyle applications to ensure that they are backed up by scientific research and that personal data are used respectfully. It is also important that users have knowledge of the state of the art and understand the current risks and limitations, so they can use these tools with the right confidence.

## Author contributions

SPM and SI contributed equally to the conception, design, and execution of this review article, as well as the drafting and revising of the manuscript. SPM played a significant role in the conceptualization of the review article, including the identification of the research questions and objectives, conducted an extensive literature review, critically analyzed the relevant studies, and synthesized the key findings, and contributed equally to the writing and editing of the manuscript, ensuring its coherence, clarity, and scientific accuracy. SI made substantial contributions to the overall design and structure of the review article, providing valuable insights and expertise in the field, actively participated in the literature review process, critically evaluating the selected studies and contributing to the synthesis of the findings, and equally contributed to the writing and revision of the manuscript, ensuring the accuracy and quality of the content. All authors collaborated closely throughout the entire research process, discussing and refining ideas, analyzing data, and making joint decisions regarding the content and scope of the article, read and approved the final version of the manuscript and take joint responsibility for its content.
